# Enhancing legacy in palliative care: study protocol for a randomized controlled trial of Dignity Therapy focused on positive outcomes

**DOI:** 10.1186/s12904-015-0041-z

**Published:** 2015-09-21

**Authors:** Lori P. Montross-Thomas, Scott A. Irwin, Emily A. Meier, Jarred V. Gallegos, Shahrokh Golshan, Eric Roeland, Helen McNeal, Diane Munson, Laura Rodseth

**Affiliations:** San Diego Moores Cancer Center, Psychiatry & Psychosocial Services; Patient & Family Support Services, University of California, 9500 Gilman Drive #0664, La Jolla, San Diego, CA 92093-0664 USA; San Diego Department of Psychiatry, University of California, 9500 Gilman Drive #0664, La Jolla, San Diego, CA 92093-0664 USA; San Diego Department of Family & Preventive Medicine, University of California, 9500 Gilman Drive #0664, La Jolla, San Diego, CA 92093-0664 USA; University of San Diego, San Diego, USA; San Diego Moores Cancer Center, Doris A. Howell Palliative Care Service, University of California, San Diego, USA; California State University Institute for Palliative Care, San Diego, USA; Family Health Centers of San Diego, San Diego, USA; Mission Hospice, San Diego, USA

**Keywords:** Cancer care, Hospice care, Palliative care, Dignity therapy, End-of-life care, Life satisfaction, Quality of life, Positive outcomes

## Abstract

**Background:**

Dignity Therapy is a brief psychotherapy that can enhance a sense of legacy while addressing the emotional and existential needs of patients receiving hospice or palliative care. In Dignity Therapy, patients create a formalized “legacy” document that records their most cherished memories, their lessons learned in life, as well as their hopes and dreams for loved ones in the future. To date, this treatment has been studied for its impact on mitigating distress within hospice and palliative care populations and has provided mixed results. This study will instead focus on whether Dignity Therapy enhances positive outcomes in this population.

**Methods/Design:**

In this study, 90 patients with cancer receiving hospice or palliative care will complete a mixed-methods randomized controlled trial of Dignity Therapy (*n* = 45) versus Supportive Attention (*n* = 45). The patients will be enrolled in the study for 3 weeks, receiving a total of six study visits. The primary outcomes examine whether the treatment will quantitatively increase levels of positive affect and a sense of life closure. Secondary outcomes focus on gratitude, hope, life satisfaction, meaning in life, resilience, and self-efficacy. Using a fixed, embedded dataset design, this study will additionally use qualitative interviews to explore patients’ perceptions regarding the use of positive outcome measures and whether these outcomes are appropriately matched to their experiences in therapy.

**Discussion:**

Dignity Therapy has shown mixed results when evaluating its impact on distress, although no other study to date has solely focused on the potential positive aspects of this treatment. This study is novel in its use of mixed methods assessments to focus on positive outcomes, and will provide valuable information about patients’ direct experiences in this area.

**Trial registration:**

ISRCTN91389194

## Background

Caring for cancer patients in hospice or palliative care involves addressing many physical, psychological, social, and spiritual concerns [1–4]. In doing so, there is often a two-fold mission: 1) decreasing suffering and distress while 2) increasing positive quality of life [5, 6]. For example, in order to address the psychological concerns of cancer patients receiving hospice or palliative care, several psychotherapies have been created and studied [2, 7–12].

One such therapy is Dignity Therapy; a brief, individualized psychotherapy designed to address the emotional and existential needs of adults who are receiving hospice or palliative care while enhancing a sense of legacy [13–17]. In therapy, patients complete an interview that highlights their most important memories, the times they felt most alive, and their most important accomplishments and roles. Additionally, patients are able to reflect on the lessons they have learned in life, and describe any hopes and dreams they have for their loved ones in the future. This interview is recorded, transcribed, and edited to create a formalized “legacy document” which can then be given to the patient’s family and friends as a keepsake.

The first Phase I trial of Dignity Therapy with 100 patients demonstrated significant decreases in depressive symptoms and patients’ sense of suffering at post-treatment, with the majority of patients reporting an increased sense of dignity (76 %), sense of purpose (68 %), and sense of meaning (67 %) [15]. A subsequent study of 60 family members, whose loved ones had completed Dignity Therapy, resulted in similar trends, providing corroboration that the treatment was viewed favorably and was highly recommended as a treatment for others [18].

The most recent Phase III randomized controlled trial of Dignity Therapy compared patients who received Dignity Therapy (*n* = 108), Client Centered Care (*n* = 107), and Standard Care (*n* = 111) on the primary outcomes of distress [19]. Interestingly, no significant differences were found between the 3 treatment groups on the primary measures of distress. However, on secondary measures the Dignity Therapy patients reported significantly higher ratings regarding the treatment being helpful, improving quality of life, increasing a sense of dignity, being beneficial to the family, and changing the way the family appreciated the patient. In light of the mixed results, the researchers noted the study instruments may have been unresponsive to the beneficial changes demonstrated in the Dignity Therapy group. As such, the researchers concluded that “future research exploring the beneficial effects of Dignity Therapy will help unravel the psychological, spiritual, and existential complexities for an individual facing death…” (p. 9).

We began to explore the beneficial effects of Dignity Therapy at San Diego Hospice and the Institute for Palliative Care in 2009. In this environment, we provided Dignity Therapy as a clinical service to patients with cancer and other diagnoses, serving as the first hospice in the country to do so. As a result, we gathered valuable pilot data regarding the referral processes, logistics, and basic costs of treatment as well as the characteristics of patients who completed Dignity Therapy [20]. We additionally gathered pilot data from 23 patients, 7 family members, and 18 hospice staff who were asked to rate the treatment. All three groups rated Dignity Therapy as “highly worthwhile” and “helpful.” During additional qualitative interviews, the staff did not frequently describe Dignity Therapy as a tool to mitigate distress, but spoke more often about it as a *positively enhancing experience*; one that provided a valuable sense of contentment, gratitude, hope, meaning, and resilience for patients as well as a way to address unfinished business and create a sense of connectedness among the patients, families, and friends [21].

Overall, the results of the previous randomized controlled trials as well as our pilot studies suggest that Dignity Therapy can provide positive benefits, although these outcomes have not been systematically investigated to date. Therefore this study is designed to measure the *positive impact* of enhancing legacy via Dignity Therapy among patients with cancer receiving hospice or palliative care. Specifically, this study aims to examine whether Dignity Therapy can significantly increase positive outcomes such as contentment, gratitude, hope, meaning, positive affect, satisfaction with life, and a sense of life closure. An additional line of inquiry is whether patients’ levels of resilience and self-efficacy play a mediating or moderating role in the treatment’s impact.

Moreover, patients in this study will be asked to share their direct experience with the therapy, and will be asked to provide their feedback about the applicability of positive outcome measures. Using a mixed-methods design, the study incorporates quantitative treatment outcomes in addition to qualitative data regarding patients’ perceptions. These results can guide future positive outcome research, particularly as we aim to design studies that fit patients’ needs.

## Methods and design

**Fig. 1:**
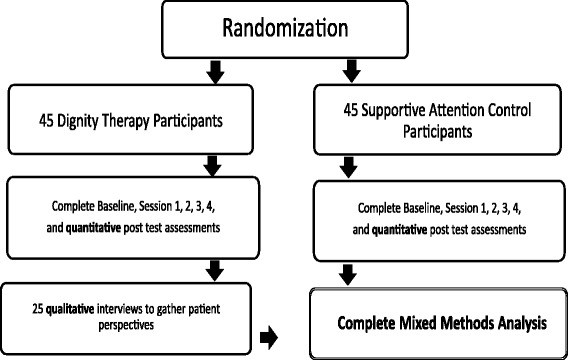
Study flow-chart of the randomized controlled trial of Dignity Therapy focused on positive outcomes

This study involves a mixed-methods randomized controlled trial of Dignity Therapy versus a Supportive Attention control group (please see Fig. 1 for an overview of the study activities). Specifically, this study involves providing either Dignity Therapy or Supportive Attention to 90 patients receiving hospice or palliative care who have a primary diagnosis of cancer. The aim is to assess whether patients who receive Dignity Therapy will exhibit significantly greater contentment, hope, gratitude, positive affect and sense of closure, and whether patients’ levels of resilience and self-efficacy mediate or moderate this effect.

All of the patients in the study will be followed for 14 days, with evaluations occurring at baseline and at the end of treatment. Each hour-long therapy session also includes the administration of two brief assessments related to positive affect and life satisfaction to explore whether each session provides some level of beneficial effect (please see Table 1 for a list of the study assessments and schedule). This study protocol and all accompanying materials have been approved by the University of California, San Diego Institutional Review Board and Human Research Protections Program.

**Table 1:** Study outcomes and assessment schedule for the randomized controlled trial of Dignity Therapy focused on positive outcomes

Instrument	Time in minutes	Baseline	Session 1	Session 2	Session 3	Session 4	Post-Test
Positive and Negative Affect Scale (PANAS)	5	•	•	•	•	•	•
Life Closure Scale (LCS)	10	•					•
Hearth Hope Index (HHI)	5	•					•
Satisfaction with Life (BPFSS)	3	•	•	•	•	•	•
Gratitude Questionnaire (GQ-6)	5	•					•
Connor-Davidson Resilience Scale (CD-RISC)	7	•					•
Hospital Anxiety and Depression Scale (HADS)	5	•					•
Life Evaluation Questionnaire (LEQ)	10	•					•
Self-Efficacy (GSE)	5	•					•
Study Completion Questionnaire	5						•
	TOTAL	70	10	10	10	10	75

### Study sample

This study will sample 90 cancer patients receiving hospice or palliative care. These 90 participants will be randomized to either Dignity Therapy (*n* = 45) or Supportive Attention (*n* = 45). Of the 45 patients completing the Dignity Therapy, 25 randomly-selected participants will be asked to complete qualitative interviews regarding their perspectives on the treatment as well as the study’s use of positive outcomes.

#### Inclusion criteria

- Primary diagnosis of cancer
- Age ≥ 18
- Enrolled in hospice or palliative care
- Willing and able to give informed consent to participate
- Able to speak and understand English
- Able to sustain attention and effort for approximately 1 hour of interaction

#### Exclusion criteria

- Current diagnosis of severe dementia, delirium, or other cognitive impairment
- Unable to speak and understand English

After providing informed consent, patients will be randomly assigned to the Dignity Therapy or a Supportive Attention Control group. All patients will continue to receive standard hospice or palliative care with their usual physicians and care teams during the trial. This includes access to social workers and spiritual counselors if desired for any psychosocial distress.

### Study treatments

#### Dignity Therapy

Dignity Therapy is a brief, empirically-supported, individualized psychotherapy designed for adults receiving hospice or palliative care. In this study, the therapy will be performed by trained Master’s level mental health counselors who will meet with patients during 4 home-based visits over the course of 3 weeks, each session lasting approximately 1 hour. The first session will involve a consultation to evaluate the patient’s motivation for therapy and to determine any specific goals they have for creating the legacy document. The second session will involve the completion of a life reflection interview using the standardized interview protocol [13, 15, 17, 19]. The life reflection interview will be digitally recorded, transcribed, and edited.

In the third session, the typewritten document will be read to the patient in its entirety. This session fosters autonomy by allowing the patient to provide personal edits as necessary, and ensures accuracy of content. Additional therapeutic value is gained since the reading provides an emotional reminder of the patient’s cherished memories and verifies that his/her history has been witnessed and recorded according to his/her wishes. The final session will then be conducted after all of the patient’s edits have been completed and formatted into a bound document. In this session a hard-copy version of the “legacy” document will be given to the patient and provided for dissemination to loved ones as a keepsake if desired.

#### Supportive attention

Patients assigned to the Supportive Attention control group will be paired with a trained Master’s level mental health counselor. These counselors will provide attention, a compassionate presence, and address any of the patient’s here-and-now concerns, but will not focus on enhancing a sense of legacy. In order to mirror the Dignity Therapy treatment, patients in Supportive Attention will also receive 4 one-hour sessions, each provided in the home, with all treatments being completed over the course of 3 weeks.

### Study outcomes

This trial will focus on the measurement of positive outcomes related to Dignity Therapy. All tests will be administered according to published standardized procedures by raters trained to a high level of inter-rater reliability (≥0.90). The tests in the battery were selected with several factors in mind: (a) validity and reliability; (b) relevance to the literature and prior experience with these tests in previous studies; and (c) limiting the length of the battery to a total of 70 minutes or less in order to reduce fatigue and undue burden for patients.

#### Primary outcome measures

##### Positive and Negative Affect

The Positive and Negative Affect Schedule (PANAS) will be used to quantify affective states. The PANAS is a 20-item self-report measure with 10 positive items and 10 negative items, thereby allowing for simultaneous measures of both positive and negative states among patients in the study. Higher scores on the 10 items in the Positive Affect scale will indicate pleasurable engagement and satisfaction with the environment, and higher scores on the Negative Affect scale will indicate anxiety, disengagement, distress, and dysfunction.

Patients will rate their agreement with emotional adjectives such as “Interested” or “Scared” on a Likert scale from 1 to 5 (1 = very slightly or not at all; 5 = extremely). The instrument will be used to measure treatment effects *within* a session as well as overarching measurements of change before and after the full intervention [22, 23].

##### Life Closure

The Life Closure Scale (LCS) is a 20-item scale to assess psychological adaptation and psychological well-being in patients at the end of life. The LCS contains two factor-analyzed subscales: Contentment and Contention. On the LCS, patients will report their level of agreement with statements using a 5-point Likert scale items such as “I can find something cheerful to think about”, “I have happy memories to help me”, or “Nothing has worked out right for me” [24].

#### Secondary outcome measures

##### Depression and anxiety

The Hospital Anxiety and Depression Scale (HADS) is a 14-item self-report measure of the relative frequency of depressed and anxious symptoms over the past week. Patients will rate items such as “I feel tense or wound up” and “I feel as if I am slowed down” on a 4-point Likert scale ranging from 0 to 3 (0 = not at all; 3 = very often) [25, 26].

##### Gratitude

The Gratitude Questionnaire Six-Item Form (GQ-6) is a brief scale designed to measure peoples’ experience of gratitude, forgiveness and spiritual transcendence. Patients will report their level of agreement with statements using a 7-point Likert scale (1 = Strongly Disagree; 7 = Strongly Agree). Sample items include: “As I get older I find myself more able to appreciate the people, events, and situations that have been part of my life history,” and “I have so much in life to be thankful for” [27].

##### Hope

The Hearth Hope Index (HHI) is a 12-item scale to assess levels of hope among people with life-threatening illnesses with items such as “I have plans for the future” and “I believe each day has potential.” Scores range from 12 to 48, with higher scores denoting greater hope. The HHI contains 3 factors assessing 1) temporality and future, 2) inner positive readiness and expectancy, and 3) interconnectedness [28].

##### Meaning in Life

The Life Evaluation Questionnaire (LEQ) is a 44-item measure of how patients with incurable cancer reflect upon their lives. Sample items include: “On the whole, life has treated me well,” and “Something good has come out of my illness.” The LEQ contains five subscales: freedom versus restriction, appreciation of life, contentment, resentment, and social integration. The 5-item Appreciation of Life subscale, the 8-item Contentment subscale and the 8-item Social Integration subscale will be used in this study [29].

##### Resilience

The Connor-Davidson Resilience Scale (CD-RISC) is comprised of 25 items using a 5-point Likert scale. The CD-RISC measures how people have felt in the past month, and scores range between 0 and 100; higher scores indicating greater resilience. Sample items include: “I tend to bounce back after illness, injury, or other hardships,” and “I give my best effort no matter what the outcome may be.” The items pertain to concepts such as personal competence, a tolerance of negative affect, a positive acceptance of change, and tenacity. This scale is used to identify whether resilience is a mediating or moderating variable among patients in the study [30].

##### Satisfaction with life

The Behavioral Risk Factor Surveillance System (BRFSS) Questionnaire was established in 1984 by the Centers for Disease Control and Prevention (CDC). The questions in this interview are now asked of more than 350,000 adults per year in all 50 U.S. states. One item within the BRFSS pertains to overall satisfaction with life. Patients in this study will state their level of agreement with this item that asks, “In general, how satisfied are you with your life?” [31].

##### Self-efficacy

The General Self-Efficacy Scale (GSE) is a 10-item scale measuring optimistic self-beliefs such as coping with adversity, persistence, or the ability to perform difficult tasks. Scores range from 10 to 40 using a 4-point scale (1 = not at all true; 4 = exactly true). Sample items include, “I can usually handle whatever comes my way” and “When I am confronted with a problem, I can usually find several solutions.” This scale is used to identify whether self-efficacy is a mediating or moderating variable for patients in the study [32].

##### Study completion questionnaire

To gather patient feedback regarding the impact of the therapy sessions, a researcher-derived questionnaire has been created for this study. The questionnaire contains 30 brief Likert-scale items for patients to rate, at the completion of the study, whether the treatment they received (either Dignity Therapy or Supportive Attention) was helpful, worthwhile, and enhanced their sense of personhood. Sample items include, “Participating in this study has helped me feel like a person, not just a disease,” and “The process of completing this study was worthwhile.”

#### Qualitative interview

As part of this study’s mixed-methods design, a randomly-selected subset of Dignity Therapy patients will complete a qualitative interview in addition to the quantitative measures described above. This one-hour qualitative interview will include 7 semi-structured questions asking patients for feedback regarding their Dignity Therapy experience - what worked, what did not, and what may have surprised them about the process. Patients will also be asked whether measuring positive outcomes is “in sync” with their experience. To this end, sample questions from the qualitative interview include: “When future researchers are interested in these types of ‘positive changes,’ what types of questions would you suggest they ask?” and “In your opinion, were there positive or negative changes we *should* have asked about as you completed your legacy document, but did not?” These data will help shape future positive outcome studies – ones that can be based directly on patients’ perspectives in hospice and palliative care.

### Statistical analyses

Descriptive statistics will be obtained for all variables, including distributions, means, medians, variances, standard deviations, skewness, kurtosis, ranges, and quartiles. Tests of normality of continuous measures will be made using the Shapiro-Wilk W and the Kolomogorov D statistics in conjunction with plots of the distribution of data and descriptive measurements. The data will also be examined for homogeneity of variance. An appropriate statistical method will be employed to correct for any abnormalities (such as log, square root, and inverse). All statistical tests will be two-tailed. Differences will be considered statistically significant provided a *p*-value ≤ 0.05 is obtained. The comparability of the two treatment groups in baseline demographic and clinical features will be tested with analyses of variance (ANOVAs) for continuous variables and Chi-square analyses for dichotomous variables.

Analyses will be based on a Random Regression Model (RRM) for continuous data using a generalized linear model [33, 34]. This model was selected over more traditional analytic approaches such as a change score, end-point, or repeated measures analysis of variance for several reasons. First, a 14–20 % death rate is anticipated based on pilot studies, and using this statistical method, we will be able to include all of the patients with missing data, early termination, or death without relying on data imputation procedures. Second, both fixed and time-varying covariates and systematic person-specific deviations from the average time trend are allowed in this method. A fully saturated treatment by time model will be utilized for inference. Co-variance structure will be chosen based on Akaikes Information Criterion (AIC). Random group level treatment effects will also be evaluated for importance based on the model AIC. This allows for any group level effects to be incorporated into the model. Denominator degrees of freedom will be calculated using the Kenward-Roger small sample correction.

In order to assess how resilience and self-efficacy may mediate or moderate the impact of Dignity Therapy, we will use a RRM method similar to the primary hypotheses. The effect of mediator and moderator variables will be explored using RRM for each candidate mediator and moderator as a main effect and an interaction [35, 36]. If the interaction effect is significant, pairwise comparisons of groups will be performed. These analyses will examine interactions of treatment group with time at each level of the mediator and moderator, and evaluate treatment levels within each subgroup.

Finally, the time course of treatment of each individual will be modeled by projecting the outcome measures on orthogonal linear, and quadratic of time. Model diagnostics will be used to determine the suitability of an autoregressive error component and nonlinear effects for assessment time. Analyses will be performed using the SPSS (version 21.0) procedures. Data will be included for all patients completing at least one assessment after baseline.

The assumptions for sample size and power calculation are based on our pilot data and using procedures described by Hedeker et al. for Random Regression Models (RRM) [37]. With the proposed sample size of 86 subjects without attrition or 90 subjects adjusted for attrition (45 for each of the two groups), the study will have power of 80 % to yield a statistically significant result for a medium effect size. Here, medium effect size is defined as a between-group difference increasing linearity from 0 at baseline to 0.5 SD units at session 4. Results from this study will provide information regarding appropriate power calculations to further determine necessary effect sizes for larger-scale studies in the future.

### Qualitative analyses

Using a methodology of Coding Consensus, Co-occurrence, and Comparison, rooted in Grounded Theory, a qualitative analysis of interviews among 25 randomly-selected patients completing Dignity Therapy will be completed [38–40]. These one-hour, semi-structured interviews will be recorded and transcribed, then two study investigators will independently read a randomly-selected 20 % subset of the interview transcripts. The initial themes present will be discussed and compared. A coding matrix will then be devised and applied to subsequent transcripts until saturation is achieved. Upon saturation, the coding matrix will be applied to all the study transcripts in their entirety with coding being completed at the paragraph level. During this coding process, disagreements in assignment or description of codes will be resolved through a discussion between the investigators and an enhanced definition of codes. A detailed analysis and calculation of the frequencies of codes and nodal relationships will be completed using Dedoose software.

## Discussion

This study will measure the positive outcomes among cancer patients in hospice and palliative care who receive Dignity Therapy versus those who receive Supportive Attention. Although Dignity Therapy is designed to address the emotional and existential needs of patients, little is known about how the treatment may increase positive aspects such as contentment, gratitude, hope, meaning, positive affect, sense of closure, and satisfaction with life.

This study addresses these questions, and utilizes a novel mixed-method design to additionally gather qualitative data regarding patients’ personal experiences with Dignity Therapy. These qualitative interviews will help researchers better understand whether positive measures are viewed as meaningful or relevant to patients, thereby leading to refined positive outcome studies in the future.

In sum, this study helps address critical questions pertaining to positive psychotherapeutic outcomes in cancer care via an emphasis on increasing patients’ sense of legacy. It has been noted that patients in today’s world of health care resonate more strongly with how healthcare can *enhance* their experience and quality of life versus only focusing on how treatments may diminish pain or suffering [41]. Randomized controlled trials that systemically measure the *beneficial* impacts of treatments are needed now and in the future in order to fortify the hospice and palliative care response to these important patients’ needs.

## References

[1] Chochinov HM, Cann BJ (2005). Interventions to enhance the spiritual aspects of dying. J Pall Med.

[2] Engelberg RA, Downey L, Wenrich MD, MD, Carline JD, Silvestri GA, Dotolo D, et al. Measuring the quality of end-of-life care. J Pain Symptom Manage. 2010;39(6):951–71. 10.1016/j.jpainsymman.2009.11.313 20538181

[3] Ferris FD, Balfour HM, Bowen K, Farley J, Hardwick M, Lamontagne C, et al. A model to guide patient and family care: based on nationally accepted principles and norms of practice. J Pain Symptom Manage. 2002;24(2):106–23. 10.1016/s0885-3924(02)00468-2 12231127

[4] Sutton LM, Porter LS, Keefe FJ (2002). Cancer pain at the end of life: a biopsychosocial perspective. Pain.

[5] Payne SA, Langley-Evans A, Hillier R (1996). Perceptions of a ‘good’ death: a comparative study of the views of hospice staff and patients. Palliat Med.

[6] Costello J (2006). Dying well: nurses’ experiences of ‘good and bad’ deaths in hospital. J Adv Nurs.

[7] LeMay K, Wilson KG (2008). Treatment of existential distress in life threatening illness: a review of manualized interventions. Clin Psychol Rev.

[8] Breitbart W (2003). Reframing hope: Meaning-centered care for patients near the end of life. Interview by Karen S. Heller. J Pall Med.

[9] Breitbart W, Rosenfeld B, Gibson C, Pessin H, Poppito S, Nelson C, et al. Meaning-centered group psychotherapy for patients with advanced cancer: a pilot randomized controlled trial. Psychooncology. 2010;19(1):21–8. 10.1002/pon.1556 PMC3648880 19274623

[10] Mohr DC, Moran PJ, Kohn C, Hart S, Armstrong K, Dias R, et al. Couples therapy at end of life. Psychooncology. 2003;12(6):620–7. 10.1002/pon.746 12923802

[11] Weaver KE, Llabre MM, Lechner SC, Penedo F, Antoni MH. Comparing unidimensional and multidimensional models of benefit finding in breast and prostate cancer. Qual Life Res. 2008;17(5):771–81. 10.1007/s11136-008-9348-z 18500579

[12] Wetherell JL, Sorrell JT, Thorp SR, Patterson TL. Psychological interventions for late-life anxiety: a review and early lessons from the CALM study. J Geriatr Psychiatry Neurol. 2005;18(2):72–82. 10.1177/0891988705276058 15911935

[13] Chochinov HM (2002). Dignity-conserving care--a new model for palliative care: helping the patient feel valued. JAMA.

[14] Chochinov HM (2004). Dignity and the eye of the beholder. J Clin Oncol.

[15] Chochinov HM, Hack T, Hassard T, Kristjanson LJ, McClement S, Harlos M. Dignity therapy: a novel psychotherapeutic intervention for patients near the end of life. J Clin Oncol. 2005;23(24):5520–5. 10.1200/JCO.2005.08.391 16110012

[16] Chochinov HM, Hack T, Hassard T, Kristjanson LJ, McClement S, Harlos M. Dignity in the terminally ill: a cross-sectional, cohort study. Lancet. 2002;360(9350):2026–30. 10.1016/S0140-6736(02)12022-8 12504398

[17] Chochinov HM (2012). Dignity therapy: Final words for final days.

[18] McClement S, Chochinov HM, Hack T, Hassard T, Kristjanson LJ, Harlos M. Dignity therapy: family member perspectives. J Pall Med. 2007;10(5):1076–82. 10.1089/jpm.2007.0002 17985964

[19] Chochinov HM, Kristjanson LJ, Breitbart W, McClement S, Hack T, Hassard T, et al. Effect of dignity therapy on distress and end-of-life experience in terminally ill patients: a randomised controlled trial. Lancet Oncol. 2011;12(8):753–62. 10.1016/S1470-2045(11)70153-X PMC3185066 21741309

[20] Montross L, Winters KD, Irwin SA (2011). Dignity therapy implementation in a community-based hospice setting. J Pall Med.

[21] Montross LP, Meier EA, De Cervantes-Monteith K, Vashistha V, Irwin SA. Hospice staff perspectives on Dignity Therapy. J Pall Med. 2013;16(9):1118–20. 10.1089/jpm.2013.0030 PMC3776611 23937061

[22] Crawford JR, Henry JD (2004). The positive and negative affect schedule (PANAS): construct validity, measurement properties and normative data in a large non-clinical sample. Br J Clin Psychol.

[23] Watson D, Clark LA, Tellegen A (1988). Development and validation of brief measures of positive and negative affect: the PANAS scales. J Pers Soc Psychol.

[24] Dobratz MC (2004). The life closure scale: Additional psychometric testing of a tool to measure psychological adaptation in death and dying. Res Nurs Health.

[25] Mitchell AJ, Meader N, Symonds P (2010). Diagnostic validity of the Hospital Anxiety and Depression Scale (HADS) in cancer and palliative settings: A meta-analysis. J Affect Disord.

[26] Walker J, Postma K, McHugh GS, Rush R, Coyle B, Strong V, et al. Performance of the Hospital Anxiety and Depression Scale as a screening tool for major depressive disorder in cancer patients. J Psychosom Res. 2007;63(1):83–91. 10.1016/j.jpsychores.2007.01.009 17586341

[27] Mccullough ME, Emmons RA, Tsang JA (2002). The grateful disposition: a conceptual and empirical topography. J Pers Soc Psychol.

[28] Herth K (1992). Abbreviated instrument to measure hope: Development and psychometric evaluation. J Adv Nurs.

[29] Salmon P, Manzi F, Valori RM (1996). Measuring the meaning of life for patients with incurable cancer: The life evaluation questionnaire (LEQ). Eur J Cancer.

[30] Connor KM, Davidson JR (2003). Development of a new resilience scale: the Connor-Davidson Resilience Scale (CD-RISC). Depress Anxiety.

[31] Stein AD, Lederman RI, Shea S (1993). The Behavioral Risk Factor Surveillance System questionnaire: Its reliability in a statewide sample. Am J Public Health.

[32] Schwarzer R, Jerusalem M, Weinman J, Wright S, Johnston M (1995). Generalized Self-Efficacy scale. Measures in health psychology: A user’s portfolio Causal and control beliefs.

[33] Laird NM, Ware JH (1982). Random-effects models for longitudinal data. Biometrics.

[34] Hedeker D, Gibbons RD, Davis JM (1991). Random regression models for multicenter clinical trials data. Psychopharmacol Bull.

[35] Kraemer HC, Wilson GT, Fairburn CG, Agras WS. Mediators and moderators of treatment effects in randomized clinical trials. Arch Gen Psychiatry. 2002;59(10):877–83. 10.1001/archpsyc.59.10.877 12365874

[36] Bauman AE, Sallis JF, Dzewaltowski DA, Owen N. Toward a better understanding of the influences on physical activity: The role of determinants, correlates, causal variables, mediators, moderators, and confounders. Am J Prev Med. 2002;23(2 Suppl):5–14. 10.1016/s0749-3797(02)00469-5 12133733

[37] Hedeker D, Gibbons R, Waternaux C (1999). Sample size estimation for longitudinal designs with attrition: Comparing time-related contrasts between two groups. J Educ Behav Stat.

[38] Palinkas LA, Horwitz SM, Chamberlain P, Hurlburt MS, Landsverk J. Mixed-methods designs in mental health services research: A review. Psychiatr Serv. 2011;62(3):255–63. 10.1176/ps.62.3.pss6203_0255 21363896

[39] Williams DG, Best AJ, Taylor DW, Gilbert JR, Wilson DMC, Lindsay EA, Singer J (1992). A systematic approach for using qualitative methods in primary prevention research. Med Anthropol Q.

[40] Glaser BG, Strauss A (1967). The Discovery of Grounded Theory: Strategies for Qualitative Research.

[41] Pierson CM, Curtis JR, Patrick DL (2002). A good death: a qualitative study of patients with advanced AIDS. AIDS Care.

